# Supplementary material for: All-at-once RNA folding with 3D motif prediction framed by evolutionary information
Source: Nat Methods. 2025 Oct 3;22(10):2094–106. doi: 10.1038/s41592-025-02833-w (PMC12510878; doi:10.1038/s41592-025-02833-w)
Supplement: Supplementary file 3 — Source code to run CaCoFold-R3D. Data and results. [file 41592_2025_2833_MOESM3_ESM.gz › supplemental_material/results/CaCoFold-R3D/CaCoFold-R3D_Rfam_vshuffle/RF01174_snoU43.R2R.sto.pdf]

RF01174_snoU43

5′ A U G A U G A ● ● ● ● R ● U ● R A A ● ● U U U A U G G U U A G A A ● A Y R ○ ● U G A ● Y A A C C ● U G A U ● A U ● U ● ● ● C U A G G G C G G ● C U G A Y Y ● ●
